# Assessment of risk factors associated with multi-drug resistant tuberculosis (MDR-TB) in Gulu regional referral hospital

**DOI:** 10.4314/ahs.v23i3.41

**Published:** 2023-09

**Authors:** Kizito Omona, Albert Mucha Opiyo

**Affiliations:** 1 Faculty of Health Sciences, Uganda Martyrs University, Kampala, Uganda; 2 Department of Public Health, Victory Health Services, Gulu City, Uganda

**Keywords:** MDR-TB, tuberculosis, HIV, Gulu Regional Referral Hospital

## Abstract

**Background:**

Multi-drug resistant tuberculosis (MDR-TB) is increasingly recognized as emerging infectious disease of public health concern. Globally, 206030 people were diagnosed with MDR-TB in 2019, representing a 10% increase from 186883people who had it in 2018. In Uganda, the prevalence of MDR among new TB cases is 4.4% and 17.7% among previously treated TB cases.

**Aim:**

To determine the risk factors associated with MDR-TB among tuberculosis patients in Gulu regional referral hospital.

**Material and Methods:**

A cross-sectional analytical study using both quantitative and qualitative methods of data collection and analysis was used. Data was collected from 384 TB patients using data extraction form and 6 Key informant interviews conducted. Analysis using Pearson chi-square test was run.

**Results:**

HIV positive patients were 2.6 times more likely to be infected with MDR-TB than HIV negative patients [AOR=2.6: 95% CI 1.34– 5.85: P=0.006]. Previously treated TB patients were 2.8 times more likely to be infected with MDR-TB than newly diagnosed TB patients [AOR=2.8: 95% CI 1.33– 5.85: P=0.006]. Defaulting TB patients were 3.1 times more likely to be infected with MDR-TB than the non-defaulting TB patients [AOR=3.1]

**Conclusion:**

There is high prevalence of drug resistance among patients attending TB treatment at the facility.

## Introduction

### Background of the Study

Multi-drug resistant tuberculosis (MDR-TB) is emerging as major challenge facing tuberculosis control programs worldwide particularly in Asia and Africa. It is a challenge not only from a public health point of view but also in the context of global economy, especially in the absence of treatment for MDR-TB at national programs level in developing countries. Thus, MDR-TB has become a major public health problem and an obstacle to global TB control 1.

MDR-TB is defined as disease with mycobacterial strains that are resistant to two of the most effective and important anti TB drugs: isoniazid and rifampicin 1,2. These two drugs are considered first-line drugs and are recommended for the treatment of all individuals with drug-susceptible TB disease.

According to literature, MDR-TB is mainly due to partial or incomplete treatments, previous history of TB treatment, treatment interruption, smoking [p = 0.005, 0.025, and 0.005, respectively] 3 and HIV/TB co-infected patients [p < 0.001] 4.

Globally, 206 030 people were diagnosed with MDR-TB in 2019 and this represented a 10% increase from 186 883 in 2018 1. Overall, the 27 high burdened countries in which Uganda is among account for 85 % of all MDR-TB cases and the prevalence of MDR among new cases is 4.4% and 17.7% among previously treated TB cases in Uganda 1. MDR-TB cases are more difficult and costly to treat, in 2017, MDR TB contributed to an estimated 14% of TB deaths globally 1 as well as MDR TB accounts for a disproportionally large proportion of the financial burden for national tuberculosis control programmes.

In addition to the great global threat that the disease poses, it can also lead to the deadlier extensively drug-resistant TB (XDR-TB) which is associated with high mortality. XDR-TB is caused by *Myobacteria* that meet the same requirements as MDR-TB, but are also resistant to any fluroquinolone and to at least 1 of 3 anti-TB injectable drugs: capreomycin, amikacin, or kanamycin 5,6.

Following from similar studies in Uganda prevention of an increase in the incidence of MDR TB is therefore crucial for the success of any national tuberculosis control programme (NTP). However, there is limited literature about the risk factors for MDR TB in resource constrained setting like Uganda. Thus, ascertaining the risk factors associated with MDR TB among TB patients at Gulu regional referral hospital will be pertinent in coming up with interventions to reduce incidences of MDR-TB.

### Area of Study

Gulu regional referral hospital (GRRH), commonly known as Gulu Hospital, is located in Gulu, Northern Uganda. Gulu is the largest metropolitan area in Uganda's Northern Region. The hospital, however, serves a wide catchment area which includes the following districts: Amuru, Gulu, Kitgum, Lamwo and Pader. It is affiliated with Gulu University, where it serves as a teaching hospital for the faculty of medicine 9.

GRRH is about 343 km (213 miles), by road, north of Kampala, Uganda's capital, and largest city. GRRH is a public hospital, funded by Uganda Ministry of Health (MoH) and general care in the hospital is free. The hospital is one of the 14 regional referral hospitals in Uganda, with the capacity of 350 beds. The Standard unit of Output (SUO) is 674,146 which is fourth highest among the 14 regional referral hospitals in Uganda 9,10.

### Research Questions

The following research questions were considered;

1. What is the prevalence of MDR-TB among TB patients treated at Gulu regional referral hospital between January 2015 to December 2021?

2. What are the individual factors associated with MDR-TB among TB patients at Gulu regional referral hospital?

3. What are the health facility related factor associated with MDR-TB among TB patients at Gulu regional referral hospital?

### Conceptual Framework for the Study

**Figure 1:**
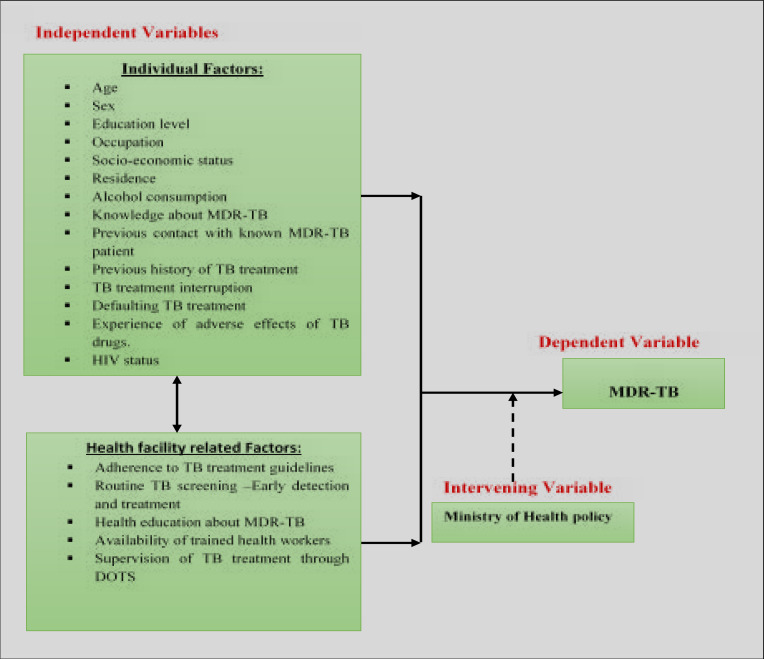
Conceptual framework diagram for the study

The conceptual framework (Figure 1) is composed of dependent, intervening and independent variables. Independent variables include Individual and health facility related factors. Individual factors included; Age, Sex, Education level, Occupation, Socio-economic status, Residence, Alcohol consumption, Knowledge about MDR-TB, Previous contact with known MDR-TB patient, Previous history of TB treatment, TB treatment interruption, Defaulting TB treatment, Experience of adverse effects of TB drugs, HIV. Health facility factors included; Adherence to TB treatment guidelines, Routine TB screening –Early detection and treatment, Health education about MDR-TB, Availability of trained health workers and Supervision of TB treatment through Directly Observed Therapies (DOTS). These inter-relate to bring about MDR-TB here described as the dependent variable for the study 2.

## Materials and Methods

### Research Design

The research design was an analytical cross-sectional study; combining both qualitative and quantitative methods.

### Study Population

The study population composed of TB patients who received treatment at Gulu regional referral hospital in period between January 2015 and December 2021 as well as health workers at the TB unit.

### Study Unit

The unit of the study involved TB patients who received treatment at Gulu regional referral hospital in the period between January 2015 and December 2021. The health workers at TB unit were also involved in the study.

### Eligibility Criteria

The study included TB patients who received treatment at Gulu regional referral hospital between January 2015 and December 2021 and excluded TB patients who received treatment at Gulu regional referral hospital outside the period between January 2015 and December 2021

### Determination of Sample Size

Sample size was determined using the Yamane formula for proportions


$$n=\frac{N}{1+N(e{)}^{2}}$$

Where

n = Sample size

N = is the population size

e = is the level of precision at a 95% level of confidence = 0.05

The cumulative number of TB patients ever treated at Gulu Regional Referral Hospital as of December 2021 was 9,492.

Therefore, from the above, the sample size shall be:


$$n=\frac{9,492}{1+9,492(0.05{)}^{2}}$$

$$n=\frac{9,492}{24.73}$$

n=384

### Sample Technique

Simple random sampling was used to select participants from the extracted list of TB patients who received treatment from Gulu regional referral hospital between January 2015 and December 2021. Purposive sampling was used to select key informants for the health facility related factors.

### Study Variables

Data variables for this study included information on number of diagnosed cases of MDR-TB, age and sex of patient, HIV status of patients diagnosed with MDR-TB, TB treatment history, and other socio-economic information correctly recorded (See table 1)

**Table 1:** Study variables and measurements

Variable	Operational Definition	Scale of measurement
**Dependent Variables**		
MDR-TB	TB patients with MDR-TB	Is the TB patient diagnosed with MDR-TB? Dichotomous (yes =1 and No-2)
**Independent variables**		
**Individual factors**		
Sociodemographic and individual characteristics of patients	Age	Numerical continuous (whole number) and Categorical ordinal (1=18-24, 2=25-29, 3=30-34, 4=35-39, 5=40-44, 6=>45)
	Gender	Categorical nominal (1=Male; 2= Female)
	Marital status	Categorical nominal (1=Married/Cohabiting, 2=Separated, 3=Widowed, 4=Single)
	Place of residence	Categorical nominal (1= Urban; 2= Rural)
	Educational level	Categorical ordinal (1= Never; 2= Primary; 3= Secondary; 4= Tertiary)
	Employment status	Is the patient employed? Categorical nominal (1=Not employed, 2=employed).
	HIV positive	Dichotomous (1= Yes; 2= No)
	Takes alcohol	Dichotomous (1= Yes; 2= No)
	Ever treated for TB	Dichotomous (1= Yes; 2= No)
	Ever interrupted TB treatment	Dichotomous (1= Yes; 2= No)
	Ever defaulted TB treatment	Dichotomous (1= Yes; 2= No)
	Previous contact of MDR-TB	Dichotomous (1= Yes; 2= No)
	Knowledgeable about MDR-TB	Dichotomous (1= Yes; 2= No)
	Ever experienced TB drugs side effects.	Dichotomous (1= Yes; 2= No)
**Health facility factors**		
Health facility factors	Routine MDR-TB screening	Interview with KIs Probe in details the answers
	Health Education on MDR-TB	Interview with KIs Probe in details the answers
	Routine TB treatment monitoring	Interview with KIs Probe in details the answers
	Availability of trained health workers.	Interview with KIs Probe in details the answers)
	Ever experienced cases of TB patients' mismanagement	Interview with KIs Probe in details the answers
	Health facility implements DOTS strategy	Interview with KIs Probe in details the answers.

### Data collection tool and method

#### Questionnaire survey

A questionnaire survey is a method of data collection containing a series of questions and providing spaces as well as options to be attempted by the respondents themselves. The questionnaire surveys used involved close-ended and open-ended questions as well as leading questions pertaining the research variables and objectives. Interview guide was used alongside this, for the key informants.

#### Pilot testing of the Instrument

A pilot study was carried out at Gulu regional referral hospital TB clinic between 14^th^ and 16^th^ December 2021. A total of 41 participants were sampled for the pre-test whom according to Otero 11, should make up more than 10% of the sample size for the actual study. During the pilot testing of the instrument, we assessed the clarity of the instruments and their ease of use. Information obtained during the pilot testing was used to revise the study instruments.

#### Data Entry, Analysis and Presentation

After data collection, the raw data collected was systematically organized to facilitate analysis. Completed questionnaires were cross examined for completeness and consistency. Descriptive statistics were used in data analysis. Data obtained from open-ended items in the questionnaires were categorized according to themes relevant to the study and were presented in a narrative form using descriptions. Analysis of data employed Statistical STA-TA 14 software where descriptive statistics were generated.

In this study, quantitative data from the questionnaires was analysed using frequency counts and frequency tables derived from the responses to the research questions. Pearson Chi Square was used to determine the existing relationship between factors associated with MDR-TB among TB patients at Gulu regional referral hospital.

#### Ethical Considerations

All the required ethical approvals were sought and granted as appropriate by Gulu regional referral hospital research and ethic committee. To ensure confidentiality, the respondents had the option to either indicate or not indicate their names on questionnaires (Voluntary participation). Informed consents were sought from each respondent.

## Results

### Demographic characteristics of respondents

From the table 2, more than two thirds 67.4% (259/384) of the study participants were males, almost a third 41% (119/384) of them were aged 45 year and above, almost a third 31.0% (119/384) had no formal education and more than two thirds 70.3% (270/384) were married.

**Table 2:** Demographic characteristics of TB patients

Variable	Category	Number/Frequency	Percentage (%)
**Sex**	Female	126	32.6
	Male	259	67.4
**Age**	18 – 24	69	18.0
	25 – 34	93	24.2
	35 – 44	103	26.8
	≥ 45	119	31.0
**Educational level**	No Education	119	31.0
	Primary	95	24.7
	Secondary	152	39.6
	Tertiary	18	4.7
**Marital status**	Married	270	70.3
	Single	114	29.7

***Source: Field data 2022***

### The prevalence of MDR- TB among TB patients at Gulu regional referral hospital

From the table 3, less than a third 22.9% (88/384) of the TB patients had MDR - TB, with 69 (17.9%) males and 19 (5.0%) females TB patients having MDR–TB respectively.

**Table 3:** Prevalence of MDR- TB among TB patients at Gulu Regional Referral Hospital

Gender	Had MDR - TB		Total

	Yes	No	
Females	19 (5.0%)	106 (27.6%)	125 (32.6%
Males	69 (17.9)	190 (49.5%)	259 (67.4%)

**Total**	**88 (22.9%)**	**296 (70.1%)**	**384 (100%)**

### Individual factors associated with MDR-TB among TB Patients

Table 4 indicated that indicate that gender (p=0.012), place of residence (p=0.001), employment status (p=0.016), HIV status of the patient (p=0.016), alcohol consumption (p=0.001), previous TB treatment (p<0.001), TB treatment interruption (p<0.001), defaulting TB treatment (p<0.001), contact with known MDR – TB case (p<0.001), knowledge about MDR – TB (p=0.005), and experience with TB drugs side effects (p<0.001) is statistically associated with MDR-TB.

**Table 4:** Pearson chi-Square Results for the individual factors associated with MDR-TB

Independent variable	Category	Had MDR-TB		Total	χ^2^(df), P-value
		No	Yes		
Sex	■ Female	106 (27.6%)	19 (5.0%)	125 (32.6%	χ^2^ (1) = 6.2473
	■ Male	190 (49.5)	69 (17.9)	259 (67.4%)	P-value = 0.012*
Age (years)	■ 18 - 24	61 (15.9%)	8 (2.1%)	69 (18.0%)	χ^2^ (3) = 6.1169
	■ 25 - 34	69 (17.9%)	24 (6.3%)	93 (24.2%)	
	■ 35 – 44	77 (20.1%)	26 (6.7%)	103 (26.8%)	P-value = 0.106
	■ ≥ 45	89 (23.2%)	30 (7.8%)	119 (31.0%)	
Place of residence	■ Rural	137 (35.7%)	23 (6.0%)	160 (41.7%)	χ^2^ (1) = 11.3286
	■ Urban	159 (41.4%)	65 (16.9%)	224 (58.3%)	P-value = 0.001*
Marital status	■ Single	91 (23.7%)	23 (6.0%)	114 (29.7%)	χ^2^ (1) = 0.6897
	■ Married	205 (53.4%)	65 (16.9%)	270 (70.3%)	P-Value = 0.406
Educational level	■ No Education	99 (25.7%)	20 (5.3%)	119 (31.0%)	χ^2^ (3) = 6.8267
	■ Primary	76 (19.8%)	19 (4.9%)	95 (24.7%)	P- Value =0.078
	■ Secondary	107 (27.9%)	45 (11.7%)	152 (39.6%)	
	■ Tertiary	14 (3.7%)	4 (1.0%)	18 (4.7%)	
Employment status	■ Employed	187 (48.7%)	43 (11.2%)	230 (59.9%)	χ^2^ (1) = 5.7844
	■ Unemployed	109 (28.4%)	45 (11.7%)	154 (40.1%)	P- Value =0.016*
HIV status	■ Negative	119 (31.0%)	23 (6.0%)	142 (37.0%)	χ^2^ (1) = 5.7592
	■ Positive	177 (46.1%)	65 (16.9%)	242 (63.0%)	P- Value =0.016*
Alcohol consumption	■ No	165 (43.0%)	31 (8.0%)	196 (51.0%)	χ^2^ (1) = 11.4255
	■ Yes	131 (34.1%)	57 (14.9%)	188 (49.0%)	P- Value =0.001*
Previously treated for TB	■ No	272 (70.8%)	59 (15.4%)	331 (86.2%)	χ^2^ (1) = 35.1990
	■ Yes	24 (6.3%)	29 (7.5%)	53 (13.8%)	P- Value <0.001*
Had TB treatment interruption	■ No	266 (69.3%)	47 (12.2%)	313 (81.5%)	χ^2^ (1) = 59.8185
	■ Yes	30 (7.8%)	41 (10.7%)	71 (18.5%)	P- Value <0.001*
Defaulted TB treatment	■ No	235 (61.2%)	32 (8.3%)	267 (69.5%)	χ^2^ (1) = 59.2811
	■ Yes	61 (15.9%)	56 (14.6%)	117 (30.5%)	P- Value <0.001*
Had contact with known MDR –TB case	■ No	292 (76.0%)	51 (13.3%)	343 (89.3%)	χ^2^ (1) = 117.7852
	■ Yes	4 (1.0%)	37 (9.6%)	41 (10.7%)	P- Value <0.001*
Knowledgeable on MDR-TB	■ No	215 (60.0%)	50 (13.0%)	265 (69.0%)	χ^2^ (1) = 7.9352
	■ Yes	81 (21.1%)	38 (9.9%)	119 (31.0%)	P- Value = 0.005*
Experienced	■ No	291 (75.8%)	79 (20.6%)	370 (96.4%)	χ^2^ (1) = 14.0766
TB drug side effects	■ Yes	5 (1.3%)	9 (2.3%)	14 (3.6%)	P- Value <0.001*

***Source: Field data 2022***

* ***statistically significant factor, df=degree of freedom***

Factors such as age, educational level and marital status were not statistically associated with MDR-TB as shown by the p-value (>0.05).

### Health facility factors associated with MDR - TB

The health facility factors were investigated using routine MDR-TB screening, treatment supervision through DOTS and routine TB treatment monitoring. Table 5 shows the results from the analysis.

From table 5, Routine TDR-TB screening (p<0.001) and implementation of DOTS (p<0.001) is statistically associated with MDR-TB. However, routine treatment monitoring is not statistically associated with MDR-TB (p=0.427).

**Table 5:** Pearson Chi-Square Results for the health facility factors associated with MDR-TB

Independent variable	Category	Had MDRTB		Total	χ^2^(df), P-value

		No	Yes		
Routine MDR-TB screening	■ No	58 (15.1%)	22 (5.7%)	80 (20.8%)	χ^2^ (1) = 59.2811
	■ Yes	238 (62.0%)	66 (17.2%)	304 (79.2%)	P- Value <0.001*

Implementation of DOTS	■ No	280	77 (20.1%)	357	χ^2^ (1) = 116.6754
	■ Yes	(72.9%)	11 (2.8%)	(93.0%)	
		16 (4.2%)		27 (7.0%)	P- Value <0.001*

Routine TB treatment monitoring	■ No	28 (7.3%)	70 (18.2%)	98 (25.5%)	χ^2^ (1) = 0.8353
	■ Yes	268 (69.8%)	18 (4.7%)	286 (74.5%)	P- Value = 0.427

* ***Statistically significant factor, df=degree of freedom***

Similar findings were obtained during the KI interviews as indicated in the quotes below;

“…… *at this hospital all TB patients are performed gene expert to detect MDR-TB and this has helped us to detect these cases early…*.” [Laboratory technologist].

“*…it is now by policy that all TB patients are screened for MDR – TB, this because the cases have been increasing for the past five years in our region especially. The clinicians have been very vigilant on MDR-TB screening…*.” [TB unit in-charge].

“……. *Implementation of DOTS improves treatment outcome since TB patients are supervised while taking their drugs on daily basis, however, our communities are poor and patients cannot afford to come daily to the clinic for treatment and this has affected too*

*much the prisoners. We just relay on the prison wardens to ensure the patient takes the drugs…*.” [TB-ward nurse in-charge].

“….. *the MDR-TB cases are admitted in the TB ward and closely monitored but also after the infectious phase they are discharged and start taking drugs from their homes*…” [TB ward nurse in-charge].

The study, as a way of controlling for confounding, subjected factors that were significant at bivariate analysis level to multivariate analysis by conducting a Multivariate Logistic Regression Analysis. The results depicting the Adjusted Odds Ratio (AOR) for each of the factors processed alongside the respective p values at a 5% level of significance were as presented in table 6.

**Table 6:** Multivariate Logistic Regression Results for the factors associated with MDR-TB

Independent Variable	Crude Odds Ratio	P-Value	95%CI	Adjusted Odds Ratio	P-Value	95%CI
Sex						
■ Male	2.0 1.0	0.014*	1.16 – 3.55	1.8 1.0	0.133	0.83 – 4.08
■ Female						
Place of residence						
■ Urban	2.4 1.0	0.001*	1.44 – 4.13	1.9 1.0	0.112	0.86 – 4.15
■ Rural						
Employment status						
■ Employed	0.6 1.0	0.017*	0.34 – 0.90	0.7 1.0	0.275	0.33 – 1.38
■ Unemployed						
HIV status						
■ Positive	21.9 1.0	0.017*	1.11 – 3.22	2.6 1.0	0.005*	1.32 – 5.62
■ Negative						
Alcohol consumption						
■ Yes	2.3	0.001*	1.41 – 3.80	2.2 1.0	0.097	0.87 – 5.53
■ No						
Previous TB treatment						
■ Yes	5.6 1.0	<0.001*	3.03 – 10.25	2.8 1.0	0.006*	1.34– 5.85
■ No						
Interrupted TB treatment						
■ Yes	7.71	<0.001*	4.40 – 13.59	2.0 1.0	0.192	0.71 – 5.63
■ No						
Defaulted TB treatment						
■ Yes	6.7 1.0	<0.001*	4.01 – 11.31	3.1 1.0	0.009*	1.34– 7.36
■ No						
Contact with MDR TB case						
■ Yes	53.0 1.0	<0.001*	18.10 – 154.96	75.2 1.0	<0.001*	21.34 – 264.65
■ No						
Knowledge on MDR TB						
■ Yes	2.0 1.0	0.005*	1.23 – 3.30	1.1 1.0	0.818	0.54 – 2.20
■ No						
Experiences TB drugs side effects.						
■ Yes	6.6 1.0	<0.001*	2.16 – 20.34	0.8 1.0	0.796	0.13 – 4.90
■ No						
Routine MDR-TB screening						
■ No	7.9 1.0	<0.001*	3.66 – 16.50	26.4 1.0	<0.001*	7.74 – 95.85
■ Yes						
Supervision of treatment through DOTS						
■ Yes	0.3 1.0	<0.001*	0.15 – 0.44	0.2 1.0	<0.001*	0.05 – 0.32
■ No						

From table 6, the HIV positive patients were 2.6 times more likely to be infected with MDR-TB than the HIV negative patients, and this was statistically significant [AOR=2.6: 95% CI 1.34– 5.85: P=0.006]. The previously treated TB patients were 2.8 times more likely to be infected with MDR - TB than the newly diagnosed TB patients and this was statistically significant [AOR=2.8: 95% CI 1.33– 5.85: P=0.006]. The defaulting TB treatment patients were 3.1 times more likely to be infected with MDR - TB than the non-defaulting TB treatment patients and this was statistically significant [AOR=3.1: 95% CI 1.34– 7.36: P=0.009]. Patients who had contact with known MDR TB case were 75.2 times more likely to be infected with MDR - TB than those who did not have contact with MDR – TB case, and this was strongly statistically significant [AOR=75.2: 95% CI 21.34– 264.65: P<0.001].

Patients who were not routinely screened for MDR TB were 26.4 times more likely to be infected with MDR - TB than those who were routinely screened for MDR–TB, and this was strongly statistically significant [AOR=26.4: 95% CI 7.74 – 95.85: P<0.001]. Patients who were on DOTS were 0.2 times less likely to be infected with MDR - TB than those who were not on DOTS, and this was strongly statistically significant [AOR=0.2: 95% 0.05 – 0.32: P<0.001].

However, the factors such as gender, place of residence, employment status, TB treatment interrupt, knowledge on MDR TB and experiencing TB drug side effects are not associated with MDR TB.

### Summary of Results

According to the study findings, MDR TB is associated with HIV positive status, previous TB treatment, defaulting TB treating and contact with MDR-TB case. HIV positive patients were 2.6 times more likely to be infected with MDR-TB than HIV negative patients [AOR=2.6: 95% CI 1.34– 5.85: P=0.006]. Previously treated TB patients were 2.8times more likely to be infected with MDR-TB than newly diagnosed TB patients [AOR=2.8: 95% CI 1.33– 5.85: P=0.006]. Defaulting TB patients were 3.1 times more likely to be infected with MDR-TB than the non-defaulting TB patients [AOR=3.1]

### Appendix 1: Data Extraction Sheet

**Table:** 

Pt code	Age	Sex	Marital status	Place of residence	Education al level	Employment status	HIV status	Takes alcohol	Ever been treated for TB	Ever interrupted TB treatment.	Ever defaulted TB treatment.	Previous contact of MDR-TB patient	Knowledgeable about MDR-TB	Ever experienced TB drugs side effect.	Routine MDR-TB screening	Patient on DOTS	Patient routinely monitored treatment
																	
																	
																	
																	
																	
																	

### Appendix 2: Interview Guide

Title of respondent……….

 

Signature of respondent………………..

1) In this hospital, do you routinely screen for TB including performing laboratory tests to detect cases early? Have you ever experienced delayed case detection of TB?

……………………………………………………………………………………….

2) Are patients on TB treatment routinely monitored? Approximately how many TB cases on treatment are monitored as per the guidelines?

……………………………………………………………………………………….

3) Do you have treatment guidelines for TB treatment? Are there incidences when TB patients were mismanaged in terms of inappropriate prescriptions?

……………………………………………………………………………………….

4) Are staff always trained or mentored on TB management and if yes, how often?

……………………………………………………………………………………….

5) Do health workers conduct health education sessions to patients about MDR-TB?

……………………………………………………………………………………….

6) Is the hospital implementing the DOTS strategy?

……………………………………………………………………………………….

## Discussion

### Prevalence of MDR- TB

It has been observed that less than a third 22.9% (88/384) of the TB patients had MDR - TB, this finding is higher as compared to the 2017 WHO anti-TB drug resistance surveillance data report, which showed that 4.1% of new and 19% of previously treated TB cases in the world are estimated to have multidrug-resistant tuberculosis.

However, the study findings agree with a study 12 which indicated that the prevalence of MDR-TB ranges between 3.3%-46.3%. Another study 13 conducted in Ethiopia reported that 33% of TB patients had MDR-TB. This disagrees with a study 14 conducted in Mali that indicated higher (62.62%) prevalence of MDR-TB and this is due to the fact that the study was conducted among previously treated TB patients yet our study involved both new and previously treated TB patients.

The observed high prevalence of the MDR-TB in the study could be due to failure to follow TB infection control measures since the majority (90.2%) of the MDR-TB cases were contacts of the known MDR-TB. In addition, given the fact that this study was conducted among TB patients attending Gulu RRH increases the risk of them contracting MDR-TB from the active cases due to cross infection that may result from poor TB infection measures within the hospital setting.

The high prevalence of MDR-TB among TB patients at Gulu RRH could be due to failure to implement Directly Observed Treatment Short Course (DOTS) strategy since it poses potential risk factors for acquisition of MDR-TB infection 15. It is well acknowledged that DOTS strategy is the best weapon to dismantle the spread of MDR-TB 6, therefore, since DOTS was not being implemented across all TB patients, this could have contributed to the high (63.9%) reported number of TB patients defaulting treatment hence subsequently led to the development of MDR-TB.

### Individual factors associated with MDR-TB

#### HIV status

HIV positive status is statistically significantly associated with MDR - TB, the study findings indicated that 26.9% of HIV positives had MDR-TB. This is agreeing with a Ugandan study 16, which found out that the prevalence of MDR-TB was 32.4% among HIV/TB co-infected patients. Another study 14 reported that 40% - 70% of HIV patients in Ethiopia are co-infected with MDR-TB.

Several other studies, including a systematic review in Europe and Ethiopia, have reported an association between HIV and MDR-TB 4, 13, 17, 17. This finding could be explained by the fact that high prevalence of TB/HIV coinfection might lead the bacteria to resist the drugs. The reason for this finding could due to the fact that HIV infected patients have a rapid disease progression and in settings where MDR-TB is prevalent, either in the general population or in the local population such as a hospital. This may subsequently lead to rapid development of a pool of drug resistant TB patients. Additionally, HIV positive people are more likely to be exposed to MDR-TB patients, due to either to increased hospitalizations in settings with poor infection control or association with peers who may have MDR-TB, including in hospital settings 19.

Furthermore, people with HIV infection progress from tuberculosis infection to active disease faster than immune competent people and drug mal-absorption in HIV infected patients, especially rifampicin and ethambutol, can lead to drug resistance and has been shown to lead to treatment failure 20.

#### Previous TB treatment

Previous TB treatment is statistically significantly associated with MDR-TB and the study findings indicated that a third (33%) of the previous TB treatment patients had MDR-TB. This is in line with the Ethiopian Study 21 which showed that more than a third (46.3%) of previous TB treatment cases had MDR-TB. Another study in Mali reported that 66.3% of the previously treated TB patients had MDR-TB 22.

The study finding is further supported by a study 18, reported that patients who had previous history of treatment for TB had 21 times higher risk of developing MDR-TB than patients who did not have a history of previous treatment for TB. The probable reason for developing MDR-TB could be due to repeated and inappropriate way of taking the medication that could result into the bacteria mutating and hence develop resistance against the drugs. In order to address this problem, effective implementation of the DOTS strategy and increasing number of institutions equipped with drug resistance tests for early detection of primary resistance is mandatory.

#### Defaulted TB treatment

Defaulting TB treatment is associated with MDR- TB, in this study almost half (47.8%) of patients with history of defaulting TB treatment had MDR-TB. This agrees with a study 23 and defaulting TB treatment results into treatment failure due to non-compliance to treatment and this increases the chances of treatment failure and hence developing MDR-TB. This is supported with a study 24, which is reported that patients who had history of pervious treatment failure was associated had increased risk of developing MDR-TB. The possible explanation for this strong association of previous treatment failure in DR-TB groups might be due to inadequate compliance by patients; lack of treatment supervision; poorer access to health-care facilities; and absence of infection control measures in clinics and hospitals.

#### Contact with MDR - TB case

Contact with MDR- TB is associated with MDR-TB infection, the study findings indicated that 90.2% of the patients who had contact with MDR-TB case were infected with MDR-TB. This agrees with a study (25), which reported that patients who had close contact with MDR-TB cases were 3.1 more likely to get infected with MDR – TB.

The reason for this finding is that TB is transmitted via close contact with an infected individual who is actively spreading the bacteria through coughing. Once inhaled, the infection is established with or without a visible primary lung lesion; lymphatic and hematogenous spread usually follows within 3 weeks of infection 26.

### Health facility factors associated with MDR-TB

#### Implementation of DOTS

Supervision of treatment by health workers through Directly Observed Treatment short course (DOTS) is associated with MDR-TB, the study findings indicated that the Odds of contracting MDR-TB was less in TB patients on DOTS. In the study, the KIs reported that the hospital failed to implement DOTS especially for TB patients who were prisoners and this could have accounted for more than half (63.6%) of the patients defaulting TB treatment and subsequently leading to MDR-TB. This agrees with a study 27 that reported lack of direct treatment observation by health workers was significantly associated with MDR-TB development.

Other studies 12; 15 also reported that lack of compliance with DOTS program were the potential risk factors for acquisition of MDR-TB infection. It is well acknowledged that DOTS strategy is the best weapon to dismantle the spread of MDR-TB 6. The probable reason for this is that when health workers directly supervise TB patients taking the drugs, there is little chance of defaulting and interruption treatment hence reducing the likelihood of contracting MDR-TB.

#### Routine MDR-TB screening

Routine MDR-TB screening is associated with MDR-TB, this because in most resource-poor countries, new case MDR TB patients are identified only after first-line therapy fails, by which time these patients could have further disseminated the disease. This agrees with the studies 28 15 which reported that non-routine MDR-TB screening is associated with increased prevalence of MDR-TB.

## Conclusion

The prevalence of drug resistance among patients attending TB treatment at Gulu regional referral hospital is 22.9%, this is quite high compared to previous national reports. The study findings has showed that the key drivers are mainly being HIV positive, previous TB treatment, defaulting TB treatment, contact with MDR–TB case, none-routine MDR-TB screening and failure to implement DOTS. Therefore, there in need for Gulu Regional Referral Hospital and the surrounding districts to promptly respond to the increasing MDR TB cases through design interventions aimed at addressing the key drivers of the disease.

## Declaration

No external funding was available for this study and the authors declare no conflict of interest.

## Authors' Information

Opiyo Albert Mucha is an MPH specialist attached to Victory Health Services, Gulu City, Uganda as the Director of the facility. His areas of interest include but not limited to HIV research, TB research and other clinical related research.

## Author infomation

Dr Omona Kizito is a Medical Doctor and Lecturer in the Faculty of Health Sciences of Uganda Martyrs University. He holds a PhD in Management (Healthcare Management), Master of Science in Health Services Management, Master of Science in Monitoring and Evaluation, Postgraduate Diploma in Project Planning and Management, and Bachelor of Medicine and Bachelor of Surgery (MBChB) from Gulu University. He is a senior researcher in the areas of Public Health, Health Services Management and Child focused research.

## Data Sharing Statement

Dataset related to this study is available with the corresponding author and can be availed on reasonable request.
